# Case Report: Recurrent cardiogenic shock caused by inter-arterial left coronary artery originating from the right coronary sinus, successfully rescued by mechanical circulatory support

**DOI:** 10.3389/fcvm.2024.1466524

**Published:** 2025-01-03

**Authors:** Tietuo Jin, Rui Wang, Liang Dong, Yvhang Lv

**Affiliations:** ^1^ Department of Intensive Care Medicine, Taizhou Central Hospital (Taizhou University Hospital), Taizhou, Zhejiang, China; ^2^ Department of Dermatology, Taizhou Central Hospital (Taizhou University Hospital), Taizhou, Zhejiang, China

**Keywords:** VA-ECMO, IABP, cardiogenic shock, AAOCA, case report

## Abstract

A young female patient suffered cardiogenic shock after undergoing surgery for an ectopic pregnancy. Coronary artery computed tomography angiography (CTA) revealed a left main artery (LM) originating from the right coronary sinus and traveling between the aorta and pulmonary artery. We successfully resuscitated the patient with mechanical circulatory support using veno-arterial extracorporeal membrane oxygenation (VA-ECMO) and an intra-aortic balloon pump (IABP). The patient subsequently underwent surgery thereafter. When sudden cardiogenic shock occurs in a young patient, it is important to be vigilant for anomalous aortic origin of a coronary artery (AAOCA).

## 1 Introduction

Anomalous aortic origin of a coronary artery (AAOCA) is the second-leading cause of sudden cardiac death (SCD) in healthy people (1). Although AAOCA has a low prevalence in the population, the left coronary artery originating from the right sinus (L-AAOCA) is even rarer, with a prevalence of only 0.02%–0.05% (2, 3). Inter-arterial left coronary artery originating from the right coronary sinus is a high-risk anatomical typing of L-AAOCA (4). Herein, we report the case of a patient with an inter-arterial left main artery (LM) originating from the right coronary sinus. In this patient, cardiogenic shock was induced by blood loss and recurred over a relatively short period of time. We successfully resuscitated the patient with veno-arterial extracorporeal membrane oxygenation (VA-ECMO) and intra-aortic balloon counter-pulsation (IABP), after which the patient underwent surgery successfully. During treatment, we observed that IABP relieved the compression of the LM that traveled inter-arterially, which has not been noted in previous studies. This case provides valuable insights into the resuscitation of AACOA-related cardiogenic shock.

## 2 Case report

The patient was a 33-year-old healthy woman who was 165 cm tall and weighed 55 kg, and she delivered a baby boy spontaneously eight years earlier. Neither she nor her family had a history of cardiac disease, and there were no signs of upper respiratory tract infection or enterovirus infection when she was admitted to the hospital. This patient was admitted to the hospital for vaginal bleeding that recurred for two months after an abortion. She was diagnosed with incomplete abortion and underwent surgery. Her hemoglobin level decreased from 11 g/L to 7 g/L due to heavy bleeding during surgery.

**Figure 1:**
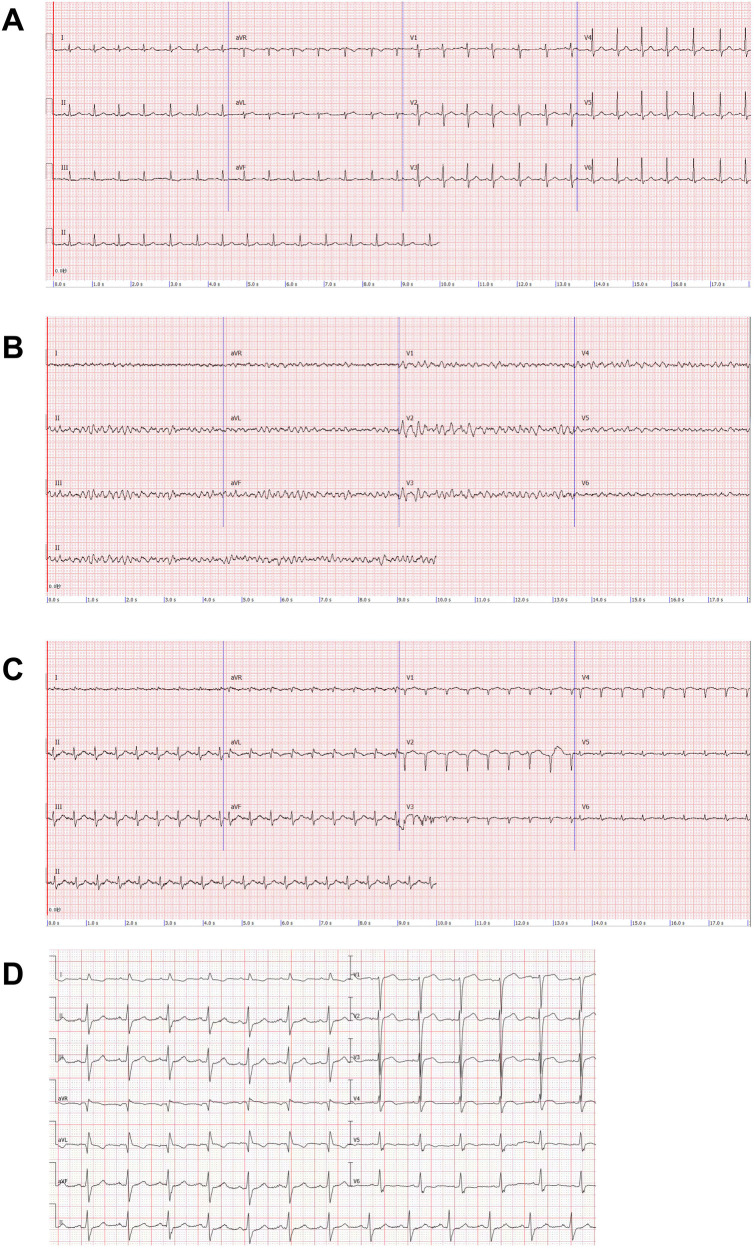
Electrocardiogram (ECG). **(A)** Patient's pre-surgical ECG. **(B)** Patient develops ventricular fibrillation after shock. **(C)** ECG after ECMO implantation: poor R-wave progression in the anterior wall leads, abnormal Q-waves in the high lateral wall leads, T-wave alterations, and a decrease in the voltage of the QRS wave in the left thoracic lead. **(D)** ECG of the patient one month after receiving unroofing.

The patient was transfused with blood cells and balanced crystalloids after surgery. Six hours later, the patient demonstrated weakness and chest tightness, with a blood pressure of 78/34 mmHg. We considered this a manifestation of hypovolemic shock; however, continuous blood transfusion and fluid resuscitation could not raise the blood pressure. Subsequently, the patient manifested respiratory and circulatory failure, and the electrocardiogram (ECG) demonstrated ventricular fibrillation (Figure 1). We immediately initiated cardiopulmonary resuscitation (CPR) and subsequently performed electrical defibrillation. After two minutes of CPR, sinus rhythm was restored and her blood pressure increased to 81/40 mmHg. After 12 min, the patient's heart rate dropped to 46/min, her blood pressure was undetectable, and her aortic pulsation disappeared; thus, we performed extracorporeal cardiopulmonary resuscitation on her. VA-ECMO (left femoral vein-right femoral artery) was implanted and the initial ECMO centrifugal pump speed was 3,490 rpm and the support flow rate was 3.36 L/min. The patient was anticoagulated with heparin, maintaining the activated partial thromboplastin time between 60 s and 80 s. A distal perfusion catheter was placed to direct a proportion of the returned oxygenated blood flow from the ECMO circuit to the distal right femoral artery. The patient's blood temperature was maintained below 36°C for cerebral resuscitation. Continuous renal replacement therapy was initiated via the ECMO circuit due to the patient's volume overload, anuria, severe metabolic acidosis, and acute kidney injury. At this time, the patient's ECG revealed myocardial ischemia (Figure 1), and the cardiac ultrasound revealed diminished myocardial contractile motion in the left ventricle with a left ventricular ejection fraction (LVEF) of 30% and a pulmonary artery pressure of 61 mmHg. The patient regained consciousness on day 2. We began administering digoxin on the fourth day of the ECMO treatment.

We attempted to make a definitive diagnosis of the patient's condition and what we could ascertain was that she did not have hypoxia, acidosis, electrolyte disturbances, and hypothermia before shock. Chest x-ray and post-CPR cardiac ultrasound did not show tension pneumothorax or pericardial effusion, and the patient did not show any signs of intoxication. Pulmonary embolism was ruled out by pulmonary artery computed tomography angiography (CTA). This patient had annual health checkups, and her electrocardiogram and cardiac ultrasound revealed no abnormalities. Epidemiologically, this patient is not at high risk of coronary heart disease. We initially considered the patient to suffer from hypovolemic shock. Shock and CPR caused the patient's myocardial injury.

**Figure 2:**
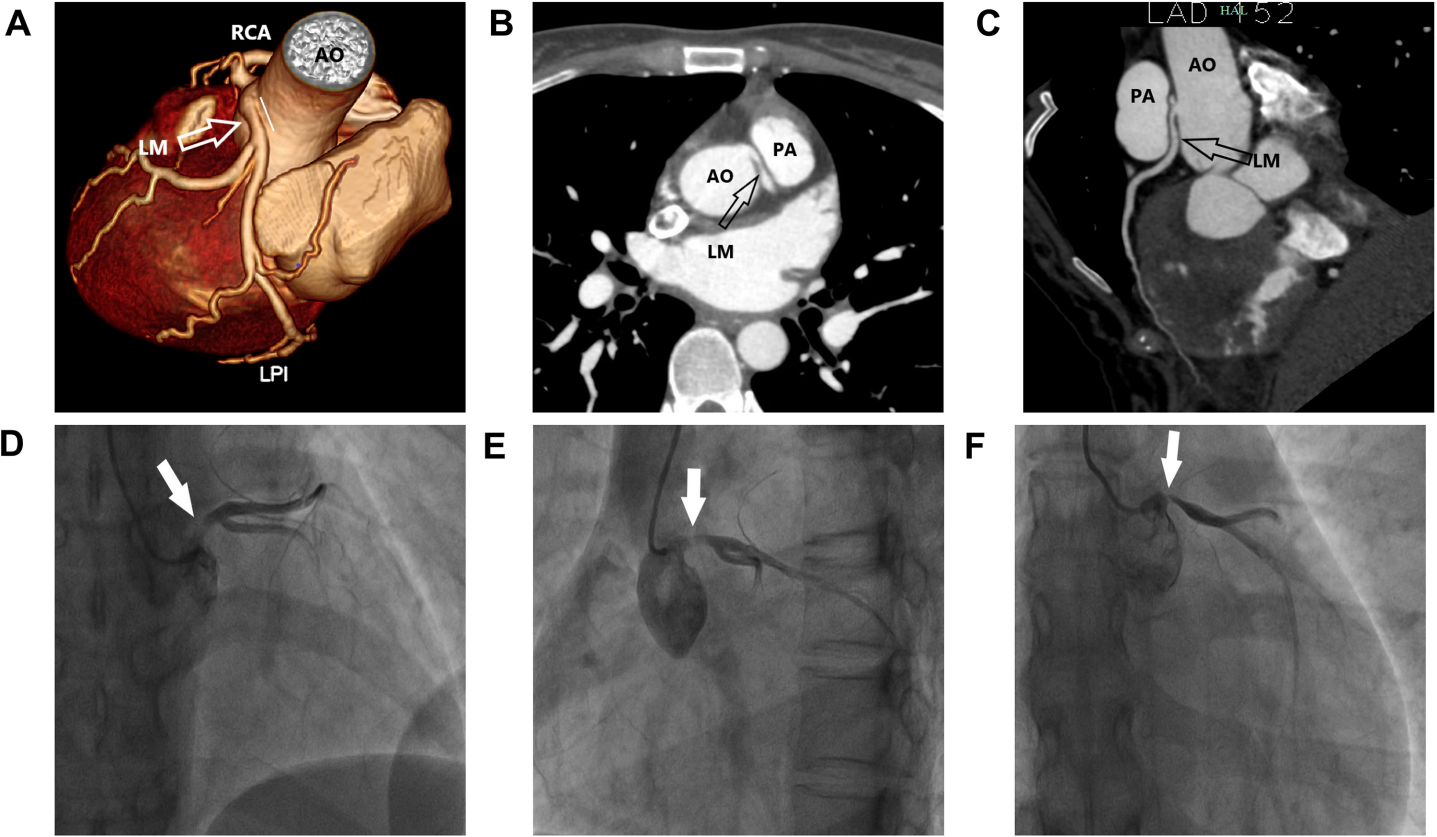
Clinical images. **(A–C)** Multiplanar reformat image showing an acute takeoff angle (<45°) of the inter-arterial left main coronary artery (LM) originating from the right coronary sinus. **(D–F)** Multi-angle coronary angiography showing LM originating from the upper part of the right coronary sinus, with an eccentric stenosis of 80%–90%.

With the help of ECMO, the patient's condition gradually improved and the dose of vasoactive drugs was gradually decreased. After seven days of ECMO, the patient's cardiac ultrasound still revealed a generalized decrease in myocardial contractile motion in the left ventricle; however, the LVEF had returned to 36% at an ECMO flow rate of 2 L/min. At this time, the patient's blood pressure was 112/55 mmHg, the dose of norepinephrine was 0.15 *µ*g/kg/min, and all her organ functions were normal except for the renal function, which had not been recovered. Therefore, we decreased the flow rate of ECMO to 1 L/min. After approximately an hour, there was no considerable change in the patient's vasoactive drugs and the ECMO was discontinued. The patient was successfully weaned from the ventilator on the third day after the ECMO was discontinued. To further investigate the etiology, we improved the coronary CTA, and the image suggested that the LM originated from the right coronary sinus and traveled between the aorta and the pulmonary artery. Coronary angiography suggested that the left coronary artery originated from the upper part of the right coronary sinus, with eccentric stenosis of 80%–90% from the opening of the LM to the proximal vessel lumen, and no notable stenosis was found in the rest of the vessels (Figure 2). Therefore, we changed the diagnosis to cardiogenic shock associated with AAOCA. The patient should have undergone surgery; however, she had poor cardiac function and was in a state of malnutrition at this time (the patient's body mass index was 15.2 kg/m^2^). We continued to provide medication, nutritional therapy, and rehabilitation. After these treatments, the patient's cardiac function and nutritional status improved. However, early in the morning of the 10th day after the first withdrawal of VA-ECMO, the patient suffered from cardiogenic shock again and developed an intermittent third-degree atrioventricular block. The patient remained conscious and her blood pressure reached a minimum of 69/43 mmHg, requiring a high dose of vasoactive amines. Her left atrium was enlarged (LA: 41 mm) and her LVEF dropped to 28%. Since the patient's cardiogenic shock was difficult to correct, she was treated with VA-ECMO again (The initial ECMO centrifugal pump speed was 3,050 rpm which supported a flow rate of 2.63 L/min).

**Figure 3:**
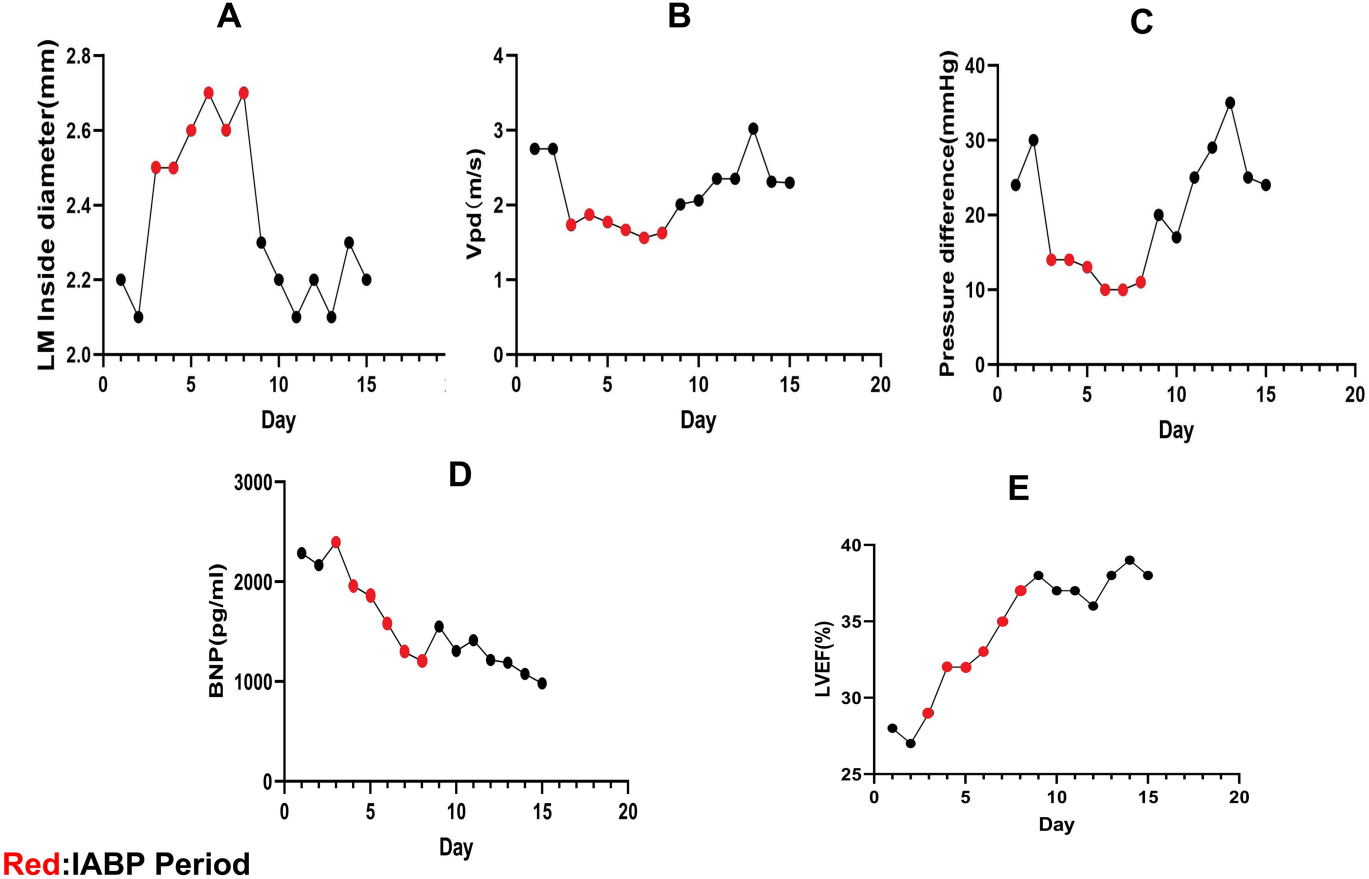
Echocardiographic data. **(A–C)** LM compression is relieved during intra-aortic balloon counter-pulsation (IABP), the LM internal diameter increases, the peak diastolic coronary flow (Vpd) velocity drops, and the LM pressure difference decreases. The LM is compressed again after stopping IABP. **(D,E)** After starting IABP therapy, this patient's brain natriuretic peptide (BNP) levels decreased and her left ventricular ejection fraction (LVEF) increased.

**Figure 4:**
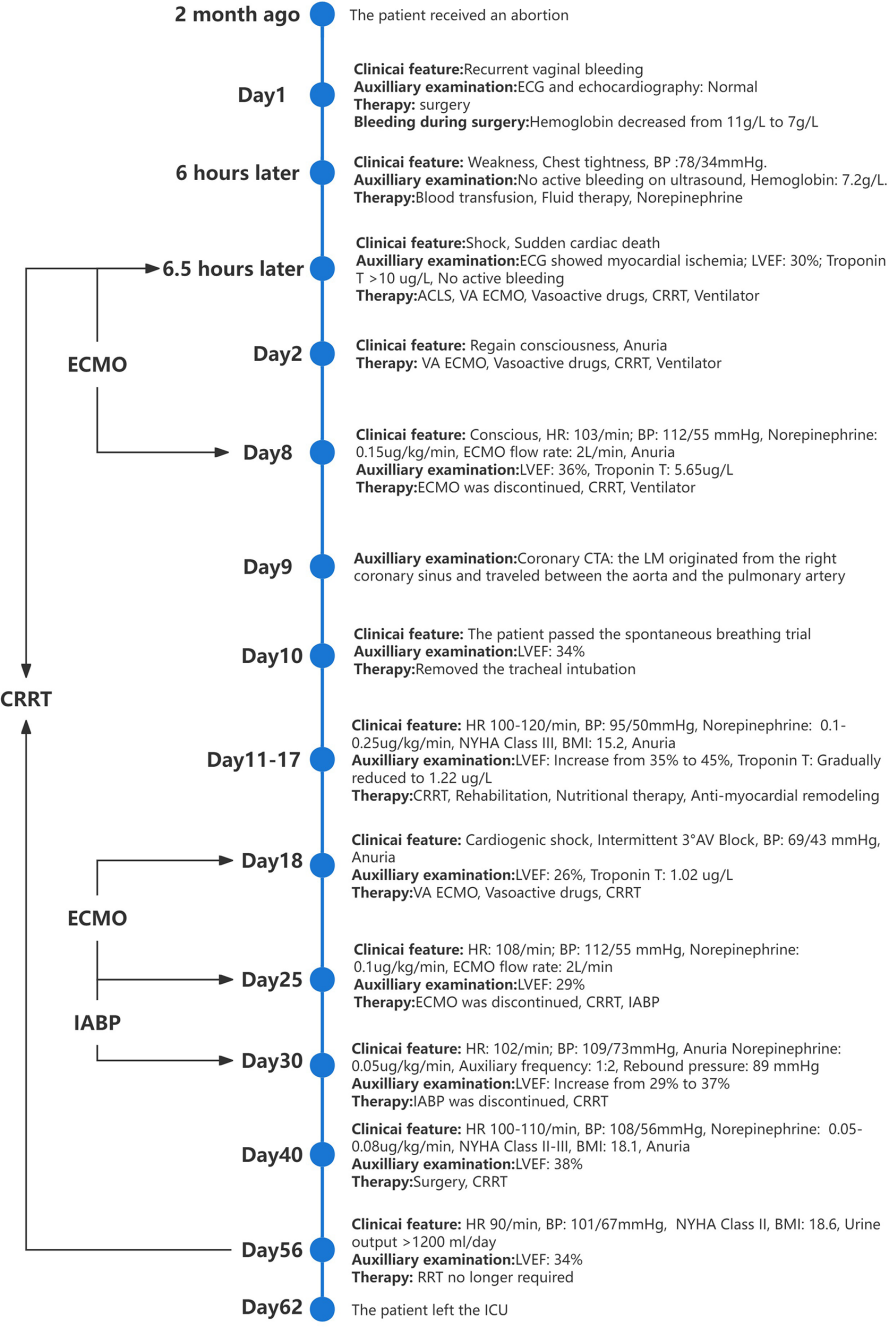
The timeline of the patient's case.

Seven days after the second VA-ECMO treatment, the patient's condition improved (her LVEF increased to 38%, her blood pressure rose to 105/65 mmHg, and the norepinephrine dose was 0.1 ug/Kg/min) and the ECMO was discontinued again. To avoid the recurrence of cardiogenic shock, we placed an IABP to support her circulation (Mode: Automatic, Trigger signal: ECG, Auxiliary frequency: 1:2, Rebound pressure: 88 mmHg). We performed echocardiography on this patient daily, and the findings suggested a decrease in the LM's lumen, a faster blood flow rate within the LM, and an increase in the pressure differential. This demonstrated that the LM was in a state of continual compression, a situation that was relieved during the period of the IABP. Also, the patient's cardiac function improved, the brain natriuretic peptide (BNP) level decreased, and the LVEF increased after IABP therapy (Figure 3). Ten days after discontinuing the IABP, we performed surgery on this patient, during which we saw the inter-arterial LM originating from the right coronary sinus and the LM within the aortic wall. She underwent unroofing of the intramural portion to relocate the LM in the appropriate sinus. In the end, the patient was in good physical and psychological condition. She was asymptomatic at rest, with minor limitations in physical activity (NYHA Class II cardiac function). Her renal function was restored on day 56, before which she had been on renal replacement therapy. Her glomerular filtration rate is currently 120 ml/min and her urine output is >1,200 ml/day. One month after receiving unroofing, the patient's ECG results suggested significant improvement in myocardial ischemia. Figure 4 summarised the patient's treatment history by way of a timeline.

## 3 Discussion

Due to the high risk of SCD with an inter-arterial left coronary artery originating from the right coronary sinus, the American College of Cardiology and American Heart Association guidelines recommend surgery even in asymptomatic patients (*Class I, Level of evidence B*) (5). The presence of a coronary artery within the aortic wall, the length of the inter-arterial coronary artery, slit-like coronary ostium, and stenosis proximal to the LM ostium are all anatomical risk factors for SCD (6, 7).

Traditionally, the inter-arterial pathway between the aorta and the pulmonary artery has been considered the primary mechanism of ischemia caused by compression between the two vessels (8). It is currently widely accepted that intramural travel in the aorta is the primary mechanism of myocardial ischemia in these patients. This is because the pressure in the coronary arteries and aorta is significantly higher than that in the pulmonary arteries (9). When the heart is in systole, the coronary arteries within the aortic wall are significantly compressed (10). During sympathetic excitation, the aortic stress increases and the coronary arteries within the aortic wall are further compressed to narrow the vessel lumen further. Also, because the left coronary artery originates from the right coronary sinus, the length of the former is increased. According to the Hagen-Poiseuille law, the smaller vessel lumen and the increased pipe length can cause a significant reduction in the coronary artery's volume flow. In this case, the patient has an inter-arterial LM originating from the right coronary sinus, and LM traveling within the aortic wall. This patient's sympathetic excitability increased significantly after massive blood loss, resulting in LM lumen compression and a decrease in blood flow through the left coronary artery. During the unroofing procedure, we found that the LM was at an acute takeoff angle to the aortic wall, and the ostium of the LM was slit-like. When these two conditions coexist, valvular coronary artery obstruction may occur (11, 12). At the same time, the oxygen-carrying capacity of the patient's blood is markedly reduced due to decreased hemoglobin levels. All these factors may have contributed to the pathogenesis of this patient's first episode of cardiogenic shock.

In a related study, a patient with a left inter-coronary artery originating from the right coronary sinus underwent an exercise test. During the test, the electrocardiogram showed myocardial ischemia; however, the patient was asymptomatic and the electrocardiogram returned to normal afterward. This patient also had no clinical symptoms in daily life, indicating that the myocardial ischemia occurred intermittently (13). It remains unclear whether such patients experience coronary artery compression when they are asymptomatic. In this case, we found through echocardiography that this patient's LM was persistently compressed despite the absence of clinical symptoms. This persistent compression may explain the recurrent cardiogenic shock observed during this patient's treatment, as LM flow was already markedly reduced, and any situation that increases cardiac workload could further aggravate myocardial ischemia.

VA-ECMO has been widely used in the treatment of cardiogenic shock (14). In this case, the patient had two episodes of cardiogenic shock within a short period, both of which resolved with VA-ECMO support. IABP is a common treatment applied to percutaneous mechanical circulatory support in acute myocardial infarction combined with cardiogenic shock (AMICS). In recent years, the IABP-SHOCK II study has suggested that IABP does not improve the thirty-day, one-year, and six-year mortality rates in patients with AMICS (15, 16). As of 2018, the European Society of Cardiology (ESC)/European Association for Cardio-Thoracic Surgery Guidelines for myocardial revascularization no longer recommend the routine use of IABP in patients with AMICS (*class III*) (17). Currently, studies focusing on the use of IABP in patients with L-AAOCA-related cardiogenic shock are rare. A case report of L-AAOCA revealed that bridging IABP could help restore cardiac function in patients who still had cardiac insufficiency after ECMO removal (18). However, the pathophysiologic mechanisms regarding the improvement of cardiac function by IABP in this type of patients are not described in the study. In our case, we bridged the IABP after the second withdrawal of VA-ECMO to help improve the patient's cardiac function and facilitate surgical treatment. During this procedure, we found (by echocardiography) that the IABP relieved the compression of the LM that traveled inter-arterially. This finding may provide experience in the resuscitation of L-AACOA-related cardiogenic shock. We still need large-sample studies to demonstrate the positive effect of IABP in inter-arterial left coronary artery originating from the right coronary sinus-related cardiogenic shock.

## 4 Conclusion

In our clinical practice, we must remain vigilant for the rare disease of AAOCA when encountering unexplained circulatory failure. Patients with inter-arterial left coronary arteries originating from the right coronary sinus are at risk of experiencing recurrent cardiogenic shock over a short period. VA-ECMO and IABP can effectively resuscitate patients with this condition, helping them regain cardiac function and creating opportunities for surgical treatment.

## Data Availability

The original contributions presented in the study are included in the article/Supplementary Material, further inquiries can be directed to the corresponding author.
